# Natural killer cell-based immunotherapy for acute myeloid leukemia

**DOI:** 10.1186/s13045-020-00996-x

**Published:** 2020-12-07

**Authors:** Jing Xu, Ting Niu

**Affiliations:** Department of Hematology, West China Hospital, Sichuan University, Chengdu, 610041 China

**Keywords:** Acute myeloid leukemia, Natural killer cells, Immunotherapy, Adoptive NK cell transfer, Chimeric antigen receptor-modified NK cells, Antibodies, Cytokines

## Abstract

Despite considerable progress has been achieved in the treatment of acute myeloid leukemia over the past decades, relapse remains a major problem. Novel therapeutic options aimed at attaining minimal residual disease-negative complete remission are expected to reduce the incidence of relapse and prolong survival. Natural killer cell-based immunotherapy is put forward as an option to tackle the unmet clinical needs. There have been an increasing number of therapeutic dimensions ranging from adoptive NK cell transfer, chimeric antigen receptor-modified NK cells, antibodies, cytokines to immunomodulatory drugs. In this review, we will summarize different forms of NK cell-based immunotherapy for AML based on preclinical investigations and clinical trials.

## Background

Acute myeloid leukemia (AML) is a clinically and genetically heterogeneous disease with unsatisfactory outcomes. Over the last few years, considerable progress has been achieved in the treatment of AML with the development and implementation of new drugs [1, 2]. However, allogeneic hematopoietic cell transplantation (HCT) has been recognized as the only way to cure AML so far and relapse remains a major problem. Novel therapeutic options aimed at attaining minimal residual disease (MRD)-negative complete remission (CR) are expected to reduce the incidence of relapse and prolong survival. Thus, immunotherapy becomes an option to tackle unmet clinical needs in AML [3, 4].

Immunotherapy has been recognized as an incredibly promising therapeutic strategy for numerous cancers [5]. The adoption of this treatment modality is based on mechanisms of immune surveillance/response and cancer escape [6]. Under physiological conditions, immune cells and substances in the immune system play pivotal roles in detecting and destroying pathogen-infected or neoplastically transformed cells. But they become less potent in cancer elimination when malignant cells display the loss of antigenicity and/or immunogenicity and are surrounded by an immunosuppressive microenvironment [6]. Thus, immunotherapy with strategies of reboosting patients’ own immune system or initiating new immune response to fight cancers has been demonstrated with the capacity of producing sustainable clinical benefits against both solid and hematological malignancies [7–9].

Natural killer (NK) cell-based immunotherapy represents one of the novel immunotherapeutic strategies recently, unleashing immune suppression of NK cells to attack various cancers [10–12]. With the progressive elucidation of NK cell immunobiology and the development of manipulative techniques, the field of NK cell-based immunotherapy in hematological malignancies has been expanding and accelerating over the past years, including adoptive NK cell transfer [13–16], chimeric antigen receptor (CAR)-modified NK cells [17–22], antibodies [23–25], cytokines [26, 27] and drug treatment [28–31]. Despite remarkable progress has been made, the application in AML is still at the initial stage. Firstly, clinical trials with results showing the efficacy and safety of these therapeutic approaches are limited, most of which are currently still in progress. Secondly, preclinical studies of NK cell-based immunotherapy are constantly emerging, in the aspect of new methodologies to utilize NK cells and strategies to enhance the response [32, 33].

Herein, in this review, we provide an overview of NK cell biology, the pathology of NK cells in AML and the recent advances in NK cell-based immunotherapy for AML based on preclinical investigations and clinical trials.

## Biology of NK cells

NK cells belong to innate lymphoid cells that contribute to immune system’s first-line defense against infections and malignant diseases [34]. They can be categorized into two subsets on the basis of surface expression levels of CD56 and CD16, as measured by the intensity of immunofluorescence. The canonical CD56^dim^CD16^+^ NK cell subset comprises around 90% of the total population in peripheral blood and exerts strong cytolytic activity through releasing cytotoxic granules containing perforin and granzymes. The rest 10% of NK cell population, known as CD56^bright^CD16^−^, is a potent producer of immunoregulatory cytokines including interferon (IFN)-γ, tumor necrosis factor (TNF)-α/β and interleukin (IL)-10 [35].

**Fig. 1:**
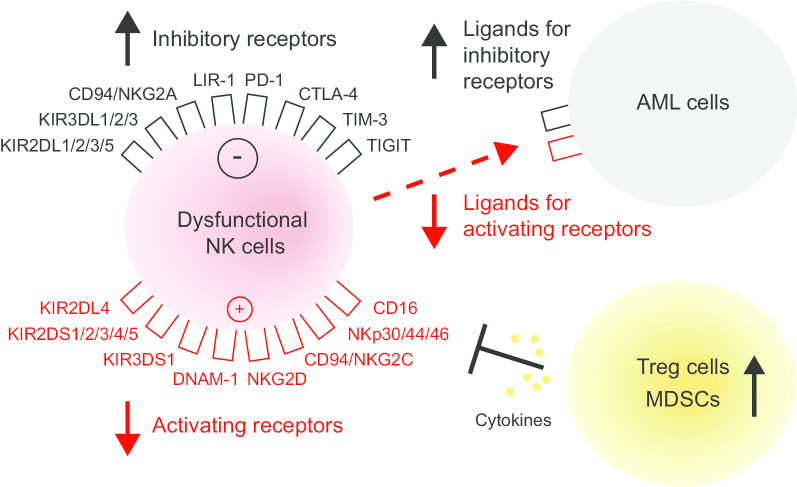
Mechanisms of immune escape from NK cell-mediated recognition in AML. Dysfunctional NK cells exhibit an imbalanced receptor expression with the overexpression of inhibitory receptors and the underexpression of activating receptors. AML cells display a defective expression of cognate ligands for NK cell activating and inhibitory receptors. The tumor microenvironment consisting of Treg cells and MDSCs can interfere with the function of NK cells through the secreting of cytokines. *MDSC* myeloid-derived suppressor cell, *NK* natural killer cell; *Treg* regulatory T cell

The cytotoxic function of NK cells is finely regulated by a complex array of surface inhibitory receptors [e.g., inhibitory killer immunoglobulin-like receptors (KIRs), leukocyte immunoglobulin-like receptors (LIRs) and CD94/natural killer group 2A (NKG2A)] and activating receptors [e.g., activating KIRs, CD94/NKG2C, NKG2D and natural cytotoxicity receptors (NCRs)] that deliver suppressive and stimulatory signals, respectively (Fig. 1) [36, 37]. In line with the diversity of major histocompatibility complex (MHC) molecules in populations, KIRs are genetically determined and display a high level of polymorphism. There are two main groups of KIR haplotypes, termed as “A” and “B”, as classified by the distinct gene content. KIR A haplotypes mainly contain inhibitory KIR genes and only one activating KIR gene KIR2DS4, whereas KIR B haplotypes carry, besides inhibitory KIR genes, various numbers and combinations of activating KIR genes [38, 39]. The considerable differences of both allelic polymorphism and KIR gene content account for the high variability of KIR gene family among different individuals.

NK cell-mediated cytotoxicity is based on the notion of “missing self-recognition” and “induced self-recognition” [40]. During NK cell development, inhibitory KIR receptors encounter with MHC class I (MHC-I) ligands on their own hematopoietic cells, leading to the acquisition of functional competence and self-tolerance [41, 42]. Both the reduction/absence of MHC-I molecules and the upregulation/de novo expression of ligands for activating receptors on tumor cells can elicit NK cell immune response against “non-self,” through releasing cytotoxic granules, secreting cytokines and inducing death receptor-dependent apoptosis [36, 43]. Apart from the direct receptor-based recognition between NK cells and tumor cells that potentiates the anti-tumor function of NK cells, they can kill tumor cells by antibody-dependent cell-mediated cytotoxicity (ADCC) as well, which is mediated by the IgG Fc receptor CD16 [44].

In addition, the activation of NK cells can be induced by other immune cells such as macrophages and dendritic cells (DCs) as well, either through direct cell-to-cell contacts or the release of cytokines such as IL-12, IL-15, IL-18 and IFN-ɑ/β, promoting NK cell cytotoxicity and IFN-γ production [45, 46].

## Dysfunction of NK cell-mediated anti-leukemia responses in patients with AML

In AML, leukemia cells can escape from NK cell-mediated recognition as a consequence of NK cell abnormalities, immunosuppressive properties of AML cells or interactions between NK cells and other immune cells in favor of immune escape (Fig. 1) [47].

Since the function of NK cells is tightly regulated by their sophisticated repertoire of inhibitory and activating receptors, imbalanced receptor expressions can lead to NK cell dysfunction. Studies evaluating the expression of these molecular receptors on NK cells showed the underexpression of activating receptors such as NKG2D, NCRs and DNAX accessory molecule-1 (DNAM-1) as well as overexpression of inhibitory receptors such as KIR2DL2/L3 and NKG2A in AML patients as compared with healthy controls [48–52]. Direct contact between AML cells and NK cells, high expression of CD200 on AML cells, soluble NKG2D ligands (NKG2DLs) in the sera and suppressive tumor microenvironment are factors that lead to defective receptor expression changes [49, 53, 54].

In addition to NK cell abnormalities, leukemia cells themselves displaying a defective expression of ligands for NK cell activating/inhibitory receptors give rise to the attenuation of NK cell-mediated anti-leukemia responses as well. For instance, the low expression of NKG2DLs [MHC class I chain-related proteins (MIC) and UL16-binding proteins (ULBP)], NCR ligands and DNAM-1 ligands (CD112 and CD155) on AML cells can render them resistant to NK cell killing [55, 56]. The deficient NKG2DL expression on AML cells may be caused by aberrant epigenetic mechanisms or the release of soluble forms from the cell surface by metalloproteinases [57, 58]. Whereas, upregulation of inhibitory immune checkpoint molecules programmed cell death ligand-1 (PD-L1) and PD-L2 is observed in AML blasts [59].

The tumor microenvironment, which possesses immunosuppressive cells, such as regulatory T cells (Tregs), myeloid-derived suppressor cells (MDSCs), tumor-associated macrophages (TAMs) and tolerogenic DCs as well as immunosuppressive factors such as transforming growth factor (TGF)-β, IL-10 and indoleamine 2,3 dioxygenase (IDO), is another major limitation to the effectiveness of NK cells in AML [60, 61].

It is worth noting that expressions of NK receptors and their cognate ligands on leukemic cells as well as the signals deriving from tumor microenvironment are deemed to impact clinical outcomes and relapse in AML patients [47]. These NK cell function-related adverse prognostic parameters including hypomaturation NK cell profile (CD56^bright^ and KIR^−^/CD57^−^), increased NKG2A and decreased NCR on NK cells, increased CD200 and decreased ULBP1 on AML cells [49, 51, 53, 62–66]. Moreover, persistence of dysfunctional NK cells was found even in patients who achieve first CR after intensive chemotherapy [67]. Thus, the presence of dysfunctional NK cells in AML and their prognostic relevance provide the rationale for the use of NK cell-based immunotherapy to restore impaired NK cell cytotoxicity against AML.

## NK cell-based immunotherapy in AML

### Adoptive NK cell transfer

**Fig. 2:**
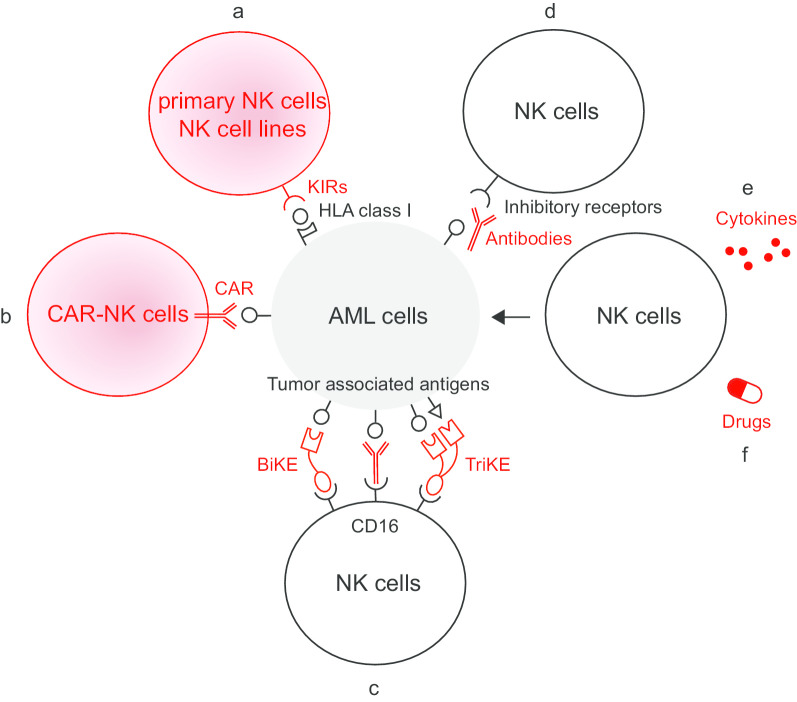
Strategies of NK cell-based immunotherapy in activating the reconstitution of NK cells against AML. **a** Adoptive NK cell transfer. **b** CAR-NK cell therapy. **c** Antibodies targeting tumor associated antigens, BiKE and TriKE. **d** Antibodies targeting NK cell inhibitory receptors. **e** Cytokines. **f** Drugs with immunomodulatory function. *BiKE* bi-specific killer cell engager, *CAR* chimeric antigen receptor, *HLA* human leukocyte antigen, *KIR* killer immunoglobulin-like receptor, *TriKE* tri-specific killer cell engager

The strategy of adoptive NK cell transfer was put forward based on beneficial effects of NK cell alloreactivity in the setting of allogeneic HCT (allo-HCT). NK cell alloreactivity is triggered by the mismatch between KIRs on donor NK cells and human leukocyte antigen (HLA) class I molecules on recipient cells, the effectiveness of which in leukemia was initially described by Perugia group [68, 69]. Alloreactions mediated by donor NK cells can kill leukemia through graft-versus-leukemia (GvL) effect, promote engraftment through ablation of recipient T cells and protect against graft-versus-host disease (GvHD) through depleting recipient antigen-presenting cells and producing IL-10 [70, 71]. Transplantation from NK alloreactive donors is considered as a strong independent factor predicting survival in allo-HCT recipients, especially from donors with more KIR B gene-content motifs [72–75]. Besides, rapid NK cell recovery post-HCT is associated with improved outcomes, while impaired NK function may be the cause of relapse [76–79]. Taken together, given the basic notions of NK cell alloreactivity and the prognostic effects of functional NK cell counts, adoptive transfer of NK cells for the management of AML has been explored in clinical applications (Fig. 2a).

Despite HCT has yielded a high rate of curability for AML, it is associated with transplant-related morbidity and mortality. Besides, not every patient is a candidate for HCT and relapse after HCT remains the most frequent cause of treatment failure. Therefore, adoptive NK cell transfer seems to be an ideal option as adjuvant and alternative treatment, and it has already been performed in the context of HCT as well as in the non-HCT setting.

#### Adoptive NK cell transfer in the context of HCT

Donor-derived NK cells are most commonly obtained from donor leukapheresis products using a magnetic cell sorting (MACS) system by CD3 depletion with or without CD56 enrichment [80–84]. They can also be generated by ex vivo differentiation from donor CD34 + hematopoietic progenitor cells [85]. NK cell transfer after HLA-haploidentical HCT is well tolerated and consolidates engraftment [80, 86]. Remarkably, a phase I study investigating the clinical effect of IL-15 plus IL-21 stimulated CD3-depleted NK cells given 2 and 3 weeks after HCT demonstrated that leukemia progression reduced compared with historical patients who have undergone HCT after the same conditioning regimen without NK cell infusion (hazard ratio 0.527, *p* = 0.042) [81]. Another phase I study showed that multiple doses of NK cells (days—2, 7 and 28 post-HCT) expanded ex vivo with K562-mbIL21-41BBL feeder cells, which were genetically modified K562 leukemia cell line expressing membrane-bound IL-21 and the 41BB ligand, could be effective in controlling leukemia relapse as well [82]. However, another study showed that compared with NK cell transfer at weeks 2 and 3 post-HCT, additional early transfer (days 6 and 9 post-HCT) was associated with significant cytokine release syndrome (CRS)-related toxicity and was not associated with less leukemia progression in patients with relapsed/refractory (R/R) AML [83]. Notably, high expression of NKp30 on donor NK cells was an independent predictor of high CR and low leukemia progression [83].

In addition, NK cells are also safe and feasible to be infused prior to HCT. A phase I study infusing escalating doses of donor-derived NK cells as a component of the preparative regimen for allo-HCT (day—8 pre-HCT) demonstrated that relapse-free survival was highly associated with the number of NK cells delivered [87]. Besides, NK cell transfer can also be applied as a bridge to HCT in R/R AML, which is useful in the reduction in disease burden to make patients eligible to proceed to HCT [84].

#### Adoptive NK cell transfer in the non-HCT setting

Since the limitations of HCT make it not applicable to all patients, it is conceivable to propel the development of adoptive NK cell transfer outside the transplantation setting.

Miller et al. was the first to conduct NK cell transfer in adult AML patients without prior HCT, reporting that haploidentical NK cell transfer with the intense high-dose cyclophosphamide and fludarabine immune suppression regimen, CD3 depletion and IL-2 administration both ex vivo and in vivo was a safe treatment with successful NK cell proliferation and activation in R/R AML (CR 5/19) [88]. Over the years, modifications to this approach have led to remarkable progress, ranging from donor selection according to KIR-ligand mismatch to improve outcomes, NK cell purification using CD3 depletion followed by CD56 enrichment to avoid side effects caused by residual cells, to milder conditioning regimens and lower dose of IL-2 in vivo to make it a well-tolerated regimen. Adoptive NK cell transfer is a feasible strategy for AML not only to induce remission, but also to maintain CR [89–93]. The combination of consolidation therapies of NK cell transfer and chemotherapy contributed to the further remission with decreased MRD and the reduction in long-term recurrence in AML patients at CR [94]. Though a phase II trial reported that NK cell transfer as a consolidation therapy for pediatric AML in first CR did not decrease relapse and increase overall survival (OS), the result of another just concluded phase II trial (NCT02763475) with a higher number of NK cell administration is worth the wait [95, 96].

Since the higher number of donor alloreactive NK cells correlates with better outcomes, ex vivo generation and in vivo expansion of an adequate number of donor NK cells with robust anti-leukemia potential are highly warranted [92]. In terms of ex vivo manipulating methods, Miller et al. demonstrated the superiority of CD3 and CD19 depletion method compared with CD3 depletion alone and CD3 depletion followed by CD56 enrichment methods, with no cause of negative effects by co-infused monocytes [97]. NK cell expansion and functional activity can be significantly enhanced by co-culturing donor's peripheral blood mononuclear cells (PBMC) with cytokines (mainly IL-2 and IL-15) or feeder cells bearing membrane-bound cytokines (such as K562-mbIL15-41BBL or K562-mbIL21-41BBL) [94, 98–100]. The feeder-free approach of using plasma membrane particles derived from K562-mbIL15-41BBL feeder cells resulted in great expansion of NK cells as well and avoided tumor-derived feeder cells being injected into patients [101]. Two phase I studies demonstrated NK cells primed with the lysate of CTV-1 leukemia cell line could prolong CR in high-risk AML patients [102, 103]. Despite IL-2 has the effect of stimulating NK cells, it stimulates host Treg cells in the meanwhile, which can inhibit NK cell proliferation and expansion in vivo. IL-15 was proposed as an alternative to IL-2 without such drawback [104, 105]. The first-in-human trial of using in vivo recombinant human IL-15 to potentiate haploidentical NK cell transfer in R/R AML showed better rates of NK cell expansion and remission compared with previous trials with IL-2, but CRS was observed when IL-15 was administered subcutaneously [106]. Furthermore, Miller et al. proposed a method of incorporating Treg depletion with IL-2 diphtheria toxin (IL2DT) into adoptive transfer platform. IL2DT was delivered to patients 1 or 2 days before NK cell transfer and it improved CR rate (53% versus 21%; *P* = 0.02) and disease-free survival (33% versus 5%; *P* < 0.01) for R/R AML patients [97]. It was showed that the use of IL2DT or low-dose irradiation as part of conditioning resulted in increased NK cell homing and persistence in the bone marrow, which correlated with better leukemia control [107].

Apart from quantity demands for NK cells, alternative sources for NK cells can facilitate their clinical applications as well. A phase I clinical trial evaluated the feasibility and safety of transferring activated human NK-92 cell lines to patients with R/R AML. NK-92 cells possess advantages of easy cultivation and expansion and can be repeatedly infused in the context of lymphodepletion [108]. Its derivative cell line NK-92MI without the presence of surface sialic acid-binding immunoglobulin-like lectins (siglec)-7 exhibited high and sustainable cytotoxicity against NK-92MI-resistant leukemia cells [109]. Besides, a study established the proof-of-concept of the feasibility of NK cells generated from CD34 + hematopoietic stem and progenitor cells (HSPC) isolated from cryopreserved umbilical cord blood (UCB) in a preclinical AML xenograft model [110]. The first-in-human study exploiting UCB-derived HSPC-NK cells in the treatment of elderly AML patients in morphologic CR found NK cell expansion and further maturation in vivo as well as a reduction in MRD without the induction of NK cell-related toxicity [111]. Another study evaluating placental-derived HSPC-NK cells (PNK-007) in R/R AML demonstrated an encouraging safety profile, but larger scale studies are needed to assess clinical outcomes [112]. A clinical trial investigating the feasibility of CYNK-001, the cryopreserved successor product to PNK-007, has recently been initiated (NCT04310592). Moreover, FT516, a NK cell product derived from a clonal master engineered induced pluripotent stem cell (iPSC) line, as a monotherapy for R/R AML is in clinical investigation (NCT04023071). These “off-the-shelf” products have significant benefits over primary NK cells from adult donors in the aspect of low costs, high therapeutic dosages, immediately application, choosing appropriate KIR B haplotype alloreactive donors and doing genetic modifications.

Further clinical trials are underway to evaluate the safety and efficacy of adoptive NK cell transfer, with the exploration of optimal NK cell dosages and resources, the optimal time points in relation to HCT and potential combination therapies. A list of currently ongoing clinical trials of NK cell transfer is provided in Table 1.

**Table 1:** Overview of ongoing clinical trials of adoptive NK cell transfer in AML

Identifier	Phase	Indication	In vivo cytokine	Transplantation	Outcome measure
PBMC-derived NK cell infusion					
NCT04221971	I	R/R AML	None	No	AE, response, NK cell metabolism, migration and reconstruction, cell count recovery, relapse
NCT04220684	I	R/R AML or MDS	None	No	MTD, AE, response, survival, cell count recovery, number of patients able to proceed to HCT
NCT04209712	I	AML with MRD	IL-2	No	MRD, AE
NCT02890758	I	AML, MDS, et al	ALT-803	No	AE, response, survival, in vivo NK level
NCT01787474 NCT02809092	I/II	R/R AML	None	No	MTD, response, NK cell expansion
NCT03300492	I/II	AML or MDS	None	Days + 10, + 15 and + 20 post-HCT	AE, survival, response, NK cell dose
NCT01823198	I/II	High-risk AML or MDS	IL-2	Day -8 pre-HCT	Optimal NK cell dose, survival, AE
NCT01904136	I/II	AML, MDS or CML	None	Days 7 and 28–90 post-HCT	MTD, AE, survival, time to engraftment
NCT04395092	II	High-risk AML or MDS	None	Days—2, + 7 and + 28 post-HCT	Relapse, AE, survival
NCT04166929	II	AML or MDS	None	Day + 7 post-HCT	Relapse
NCT03050216	II	R/R AML	ALT-803	No	Response, NK cell expansion, AE
NCT03955848	NA	AML in remission	IL-2	No	Survival
Placental-derived HSPC-NK cell (CYNK-001) infusion					
NCT04310592	I	AML	None	No	MTD, AE, MRD, survival
UCB-derived HSPC-NK cell infusion					
NCT01619761	I	AML, MDS, et al	None	Day-2 pre-CBT	AE, survival
NCT04347616	I/II	R/R AML	IL-2	No	AE, MRD, NK cell lifespan, expansion and functional activity, plasma cytokine concentration, number of patients able to proceed to HCT
NCT02727803	II	AML, MDS, et al	None	Days 30–180 post-CBT	Survival, AE
iPSC-derived NK cell (FT516) infusion					
NCT04023071	I	R/R AML or B-cell lymphoma	IL-2	No	AE, response, pharmacokinetic data
Cytokine-induced memory-like NK cell infusion					
NCT03068819	I	Relapsed AML after HCT	None	No	AE, response, survival
NCT04024761	I	Relapsed AML, MDS or MPN after HCT	IL-2	No	AE, response, survival
NCT01898793	I/II	R/R AML or MDS	IL-2	No	MTD, response, AE, survival
NCT04354025	II	R/R AML	IL-2	No	Response, number of patients able to proceed to HCT, survival, MRD, AE
NCT02782546	II	R/R AML	ALT-803	Day + 7 post-HCT	Survival, response
CMV-induced memory-like NK cell (FATE-NK100) infusion					
NCT03081780	I	R/R AML	IL-2	No	MTD, response, NK cell expansion, AE, MRD, survival

*AE* adverse event, *AML* acute myeloid leukemia, *CBT* cord blood transplantation, *CML* chronic myeloid leukemia, *CMV* cytomegalovirus, *HCT* hematopoietic cell transplantation, *HSPC* hematopoietic stem and progenitor cell, *IL* interleukin, *iPSC* induced pluripotent stem cell, *MDS* myelodysplastic syndrome, *MPN* myeloproliferative neoplasm, *MRD* minimal residual disease, *MTD* maximum tolerated dose, *NA* not applicable, *NK* natural killer cell, *PBMC* peripheral blood mononuclear cell, *R/R* relapsed/refractory, *UCB* umbilical cord blood

### CAR-NK cell therapy

In adoptive NK cell transfer, the ability of NK cells to mount an immune response against AML cells is largely dependent on the interactions between NK cell activating/inhibitory receptors with their cognate ligands on target cells. In order to augment the specificity and cytotoxicity, genetically modified NK cells such as CAR-modified-NK cells are designed (Fig. 2b). Since the success of CAR-T therapy in the treatment of B-lineage acute lymphoblastic leukemia and B-cell lymphoma has not yet been translated into the treatment of AML and its wide applications are limited by adverse effects such as CRS [113, 114], NK cells with short lifespan are being considered as promising alternatives to modified T cells with favorable toxicity profiles and low manufacturing costs [115]. Nowadays, the actions of CAR-NK cells are being extensively studied in a variety of tumor models, but the applications in AML are relatively limited and mainly at the preclinical stage.

The optimal choice of leukemia specific markers that can be targeted by CAR-NK cells is a major obstacle, since AML shares some phenotypic markers with normal hematopoietic stem cells (HSCs). Myeloid differentiation antigen CD33 is detected on blasts of > 85% of AML patients and also on leukemia stem cells (LSCs) [116]. A preclinical investigation ascertained the targeting effect of NK cell line YT with gene transfer of a CD33-specific immunoglobulin-based humanized chimeric T cell receptor (cIgTCR) to CD33 + AML cell lines [117]. The first-in-man reported phase I trial of CAR-NK cells demonstrated the safety of irradiated CD33-CD28-4-1BB-CD3ζ CAR-NK-92 cells infusion in 3 patients with R/R AML, but it did not demonstrate obvious clinical efficacy [118]. Larger-scale clinical trials are warranted to determine the effects (NCT02944162). CD4 is another antigen present on AML blasts without ubiquitous expression on HSPCs and non-hematopoietic cells. Salman et al. established the role of CD4-CD28-4-1BB-CD3ζ CAR-NK-92 cells in robustly eliminating CD4 + AML cells ex vivo and in mouse xenografts [119]. CD7 is detected in approximately 30% of AML cases and also presents as an attractive target [120, 121]. CD7-CD28-4-1BB-CD3ζ CAR-NK-92MI cells have significantly improved killing efficiency against CD7 + AML cells as compared with NK-92MI cells without genetic modifications, which provides a basis for clinical investigation (NCT02742727) [122].

As for the sources of CAR-NK cells, a preclinical study showed that CD123-CAR-NK-92 cell lines represented better CAR effector cells than primary human donor CD123-CAR-NK cells in terms of cytotoxic activities [123].

The lessons learned from CAR-T and CAR-NK cells in the treatment of other cancers are worthy to be exploited in CAR-NK cell therapy in AML in the future, including optimizing targets and structures of CAR-NK cells as well as investigating the ideal patient populations for this type of immunotherapy.

### Antibodies

In the normal physiologic setting, the interaction of receptors-ligands and the process of ADCC are involved in the NK cell activation. Taking advantage of this functionality, monoclonal antibodies become another method of boosting patients’ NK cells against AML. On the one hand, antibodies targeting tumor-associated antigens endow NK cells with the power of activation via ADCC effects. On the other hand, antibodies targeting NK cell inhibitory receptors have the potential to weaken inhibitory signals and let activating signals dominate the process. Great progress has been made in the field of antibody therapies, and the overview of ongoing clinical trials concerning novel antibodies for AML is presented in Table 2.

**Table 2:** Overview of ongoing clinical trials of antibodies for AML

Antibody	Target	Regimen	Indication	Phase	Identifier
Antibodies targeting tumor-associated antigens					
BI 836858	CD33	BI 836858 + decitabine	AML	II	NCT02632721
GO	CD33	GO + CPX-351	Relapsed AML	I	NCT03904251
		GO + venetoclax	R/R CD33 + AML	I	NCT04070768
		GO + pracinostat	R/R CD33 + AML	I	NCT03848754
		GO + allo-HCT	Average-risk CD33 + AML or MDS or JMML	I	NCT01020539
		GO, midostaurin, cytarabine and daunorubicin	Newly diagnosed FLT3 mutated AML	I	NCT03900949
		GO + talazoparib	R/R CD33 + AML	I/II	NCT04207190
		GO, midostaurin, cytarabine and daunorubicin	Newly diagnosed AML	I/II	NCT04385290
		GO, PF-04518600, venetoclax, avelumab, glasdegib and azacitidine	R/R AML	I/II	NCT03390296
		GO, G-CSF, cladribine, cytarabine and mitoxantrone	Newly diagnosed AML	I/II	NCT03531918
		GO	CD33 + AML	II	NCT03737955
		GO + allo-HCT	Average-risk CD33 + AML or MDS	II	NCT02117297
		GO + azacitidine	Newly diagnosed elderly AML	II	NCT00658814
		GO + bortezomib	R/R AML	II	NCT04173585
		GO + CPX-351	R/R CD33 + AML or high-risk MDS	II	NCT03672539
		GO + DLI	R/R AML	II	NCT03374332
		GO, mitoxantrone and etoposide	Refractory CD33 + AML	II	NCT03839446
		GO, cyclophosphamide, busulfan and allo-HCT	High-risk CD33 + AML or MDS	II	NCT02221310
		GO, fludarabine, cytarabine, filgrastim-sndz and idarubicin	Newly diagnosed AML or high-risk MDS	II	NCT00801489
		GO, daunorubicin, cytarabine and glasdegib	Newly diagnosed AML	II	NCT04168502
		GO + standard chemotherapy	Pediatric AML	II	NCT04326439
		GO + cytarabine	Newly diagnosed AML	II/III	NCT02473146
		GO + daunorubicin + cytarabine	Elderly AML	II/III	NCT02272478
		GO	Newly diagnosed AML	III	NCT04093505
		GO + standard chemotherapy	Newly diagnosed NPM1 mutated AML	III	NCT00893399
		GO + standard chemotherapy + HCT	AML	III	NCT00049517
		GO, CPX-351, gilteritinib and standard chemotherapy	Newly diagnosed AML	III	NCT04293562
		GO, liposomal daunorubicin, mitoxantrone, fludarabine, cytarabine, busulfan and cyclophosphamide	Pediatric AML	III	NCT02724163
		GO	R/R CD33 + AML	IV	NCT03727750
Lintuzumab Ac-225	CD33	Lintuzumab Ac-225, cladribine, cytarabine, mitoxantrone and G-CSF	R/R CD33 + AML	I	NCT03441048
		Lintuzumab-Ac225 + venetoclax + spironolactone	R/R CD33 + AML	I/II	NCT03867682
		Lintuzumab-Ac225 + venetoclax + azacitidine	R/R CD33 + AML	I/II	NCT03932318
Daratumumab	CD38	Daratumumab	R/R AML or high-risk MDS	II	NCT03067571
		Daratumumab + DLI	Relapsed AML after HCT	I/II	NCT03537599
Isatuximab	CD38	Isatuximab + standard chemotherapy	Pediatric R/R ALL or AML	II	NCT03860844
Magrolimab	CD47	Magrolimab + atezolizumab	R/R AML	I	NCT03922477
		Magrolimab + azacitidine	AML or MDS	I	NCT03248479
		Magrolimab + azacitidine + venetoclax	AML	I/II	NCT04435691
Cusatuzumab	CD70	Cusatuzumab, azacitidine and venetoclax	AML	I	NCT04150887
		Cusatuzumab + azacitidine	Newly diagnosed AML or high-risk MDS	I	NCT04241549
		Cusatuzumab + azacitidine	Newly diagnosed AML or high-risk MDS	I/II	NCT03030612
		Cusatuzumab + azacitidine	Newly diagnosed AML	II	NCT04023526
SEA-CD70	CD70	SEA-CD70	AML or MDS	I	NCT04227847
IMGN632	CD123	IMGN632	R/R CD123 + AML or other hematologic malignancies	I/II	NCT03386513
		IMGN632, venetoclax and azacitidine	CD123 + AML	I/II	NCT04086264
ASP1235	FLT3	ASP1235	AML	I	NCT02864290
FLYSYN	FLT3	FLYSYN	AML	I/II	NCT02789254
Atezolizumab	PD-L1	Atezolizumab + magrolimab	R/R AML	I	NCT03922477
		Atezolizumab + gilteritinib	R/R FLT3 mutated AML	I/II	NCT03730012
		Atezolizumab + guadecitabine	R/R AML, CML or MDS	I/II	NCT02935361
Avelumab	PD-L1	Avelumab, GO, PF-04518600, venetoclax, glasdegib and azacitidine	R/R AML	I/II	NCT03390296
Durvalumab	PD-L1	Durvalumab + azacitidine	Newly diagnosed MDS or elderly AML	II	NCT02775903
Antibodies targeting NK cell inhibitory receptors					
Pembrolizumab	PD-1	Pembrolizumab	Relapsed AML or MDS after HCT	I	NCT03286114 NCT02981914
		Pembrolizumab + decitabine	AML or MDS	I	NCT03969446
		Pembrolizumab + AMG 330	R/R AML	I	NCT04478695
		Pembrolizumab	Non-favorable risk AML	II	NCT02771197
		Pembrolizumab	Elderly AML in remission	II	NCT02708641
		Pembrolizumab + cytarabine	R/R AML	II	NCT02768792
		Pembrolizumab + azacitidine	NPM1 mutated AML	II	NCT03769532
		Pembrolizumab + azacitidine	R/R AML or newly diagnosed elderly AML	II	NCT02845297
		Pembrolizumab, azacitidine and venetoclax	Elderly newly diagnosed AML	II	NCT04284787
		Pembrolizumab, cytarabine, idarubicin, daunorubicin and HCT	Newly diagnosed AML	II	NCT04214249
Nivolumab	PD-1	Nivolumab	High-risk AML or MDS after HCT	I	NCT04361058
		Nivolumab	Relapsed AML after HCT	I	NCT01822509
		Nivolumab + ipilimumab	AML or MDS	I	NCT02846376
		Nivolumab + ipilimumab	High-risk R/R AML or MDS	I	NCT03600155
		Nivolumab, CDX-1401, poly ICLC and decitabine	AML or MDS	I	NCT03358719
		Nivolumab + azacytidine	Pediatric R/R AML	I/II	NCT03825367
		Nivolumab	AML in remission at high-risk for relapse	II	NCT02532231
		Nivolumab	AML in remission	II	NCT02275533
		Nivolumab, azacitidine and ipilimumab	AML	II	NCT02397720
		Nivolumab, azacitidine, midostaurin, decitabine and cytarabine	Elderly newly diagnosed AML or high-risk MDS	II/III	NCT03092674
Tislelizumab	PD-1	Tislelizumab, DNA hypomethylating agent and chemotherapy	AML	II	NCT04541277
Spartalizumab	PD-1	Spartalizumab, MBG453 and decitabine	AML or high-risk MDS	I	NCT03066648
Ipilimumab	CTLA-4	Ipilimumab	Relapsed AML after HCT	I	NCT01822509
		Ipilimumab + nivolumab	High-risk R/R AML or MDS	I	NCT03600155
		Ipilimumab + nivolumab	AML or MDS	I	NCT02846376
		Ipilimumab + decitabine	R/R AML or MDS	I	NCT02890329
		Ipilimumab + DLI	Relapsed AML, MDS or MPN after HCT	I	NCT03912064
		Ipilimumab, nivolumab and azacitidine	AML	II	NCT02397720
MBG453	TIM-3	MBG453, HDM201 and venetoclax	AML or high-risk MDS	I	NCT03940352
		MBG453, spartalizumab and decitabine	AML or high-risk MDS	I	NCT03066648
		MBG453, azacitidine and venetoclax	Newly diagnosed AML	II	NCT04150029
BiKE or TriKE					
GTB-3550	CD16/IL-15/CD33	GTB-3550	CD33 + R/R AML or high-risk MDS	I/II	NCT03214666

*ALL* acute lymphoblastic leukemia, *allo-HCT* allogeneic hematopoietic cell transplantation, *AML* acute myeloid leukemia, *BiKE* bi-specific killer cell engager, *CML* chronic myeloid leukemia, *CTLA-4* cytotoxic T lymphocyte-associated antigen-4, *DLI* donor lymphocyte infusion, *FLT3* FMS-like tyrosine kinase 3, *G-CSF* granulocyte colony-stimulating factor, *GO* gemtuzumab ozogamicin, *HCT* hematopoietic cell transplantation, *JMML* juvenile myelomonocytic leukemia, *MDS* myelodysplastic syndrome, *MPN* myeloproliferative neoplasm, *NPM1* nucleophosmin 1, *PD-1* programmed cell death-1, *PD-L1* programmed cell death ligand-1, *R/R* relapsed/refractory, *TIM-3* T-cell immunoglobulin domain and mucin domain-3, *Treg* regulatory T cell, *TriKE* tri-specific killer cell engager

#### Antibodies targeting tumor-associated antigens

Antibodies targeting tumor-associated antigens are attractive means of immunotherapy for cancers, the mechanisms of which are in great part the induction of ADCC via NK cells (Fig. 2c). The outcomes of unconjugated antibodies were generally poor when used alone [124–126]. The effects could be enhanced by engineering antibodies’ Fc parts to increase affinity to CD16 or integrating with other therapies [127–129]. Preclinical studies investigating the efficacy of novel Fc-optimized antibodies targeting various potential antigens such as CD133, CD33, CD157 and IL-1 receptor accessory protein (IL1RAP) as well as new regimens of antibodies combined with NK cell transfer exhibited promising results and these strategies can be valuable to be conducted in future clinical trials [130–136]. Antibody-drug conjugates (ADCs) and antibody-radio conjugates are promising strategies to enhance the antibody potency as well, and they yield superior clinical impacts on AML patients [137–141]. Gemtuzumab ozogamicin (GO), the combination of anti-CD33 antibody with anti-neoplastic agent calicheamicin, is currently the only ADC approved by the Food and Drug Administration (FDA) for the treatment of newly diagnosed and R/R CD33 + AML [142–144]. Latest preclinical findings of more novel ADCs targeting CD33, CD37, FLT3, C-type lectin-like molecule 1 (CLL-1; also known as C-type lectin domain family 12, member A, CLEC12A) and leukocyte immunoglobulin-like receptor subfamily B4 (LILRB4) highlight their clinical potential for the treatment of AML [145–151].

In addition, ligands of NK cell inhibitory or activating receptors on AML cells can also be the targets of antibodies. It was reported that NK-resistant feature of mixed lineage leukemia (MLL)-rearranged leukemia could be overcome by anti-CD19 antibody and anti-CD33 antibody-induced ADCC, and the effects could be further amplified with pan-MHC-I antibodies, suggesting the utilization of a triple immunotherapy approach, including KIR-mismatched NK cell transfer, antibodies against tumor-associated antigens and inhibitory KIR blockade, for the treatment of MLL-rearranged leukemia [152]. The expression level of inhibitory immune checkpoint molecule PD-L1 on AML blasts is an important negative prognostic factor [153]. Hypomethylating agents, while enhancing anti-tumor immune response, can concurrently dampen immune response by upregulating PD-1 and PD-L1 expression, providing the rationale of combination therapies of PD-L1 inhibitors and hypomethylating agents [154, 155]. Other antibodies targeting TNF family members on AML cells, such as glucocorticoid-induced TNFR-related protein ligand (GITRL) and receptor activator for NF-κB ligand (RANKL), were manifested against primary AML cells in preclinical studies through the prevention of inhibitory signals into NK cells as well as the induction of ADCC [156–158]. Despite the inevitable reduction in activating signals upon antibodies binding to ligands of activating receptors, NKG2D-Fc and NKp80-Fc fusion proteins were shown to be able to compensate for it by inducing ADCC to potentiate NK cell killing of AML cells [159, 160].

#### Antibodies targeting NK cell inhibitory receptors

Inhibitory receptors in NK cells serve as the sources of cancer immune escape, making them ideal targets for immunotherapy (Fig. 2d). Over the past decades, the number of inhibitory receptors identified in NK cells has been increasing. Apart from MHC-I-specific inhibitory receptors KIRs, LIRs and CD94/NKG2A, other immune checkpoints on NK cells have been shown to cause dysfunction such as programmed cell death-1 (PD-1), cytotoxic T lymphocyte-associated antigen-4 (CTLA-4), T-cell immunoglobulin domain and mucin domain-3 (TIM-3), T-cell immunoglobulin and immunoreceptor tyrosine-based inhibitory motif domain (TIGIT), siglec-7/9 and CD200R [161].

Just as the benefit of KIR-ligand mismatch between donors and recipients in improving the outcome of HCT, pharmacologic KIR blockade by anti-KIR antibodies can prevent the KIR-HLA-C interaction and augment NK cell function as well. IPH2101 and IPH2102 (lirilumab) are antibodies targeting KIR2D and both were reported to be safe in the treatment of elderly patients with AML in first CR, though the leukemia-free survival with lirilumab did not compare favorably to placebo in a phase II study [162–164]. The combination of lirilumab with azacitidine also did not display significant improvement in R/R AML in terms of response rate (overall response rate, ORR 14%) or survival (median OS 4.2 months), and the relevant clinical trial (NCT02399917) was terminated early due to unsatisfactory results [165]. LIR-1 or NKG2A blockade resulted in increased NK cell cytotoxicity against AML, suggesting that the cocktail consisting of anti-KIR, anti-LIR-1 and anti-NKG2A antibodies may be a necessary option for better efficacy [166, 167]. Anti-PD-1 antibody (nivolumab and pembrolizumab) and anti-CTLA4 antibody (ipilimumab) are FDA-approved immune checkpoint inhibitors mainly for the treatment of various solid tumors, while their applications in the field of AML are still at the exploratory stage. Nivolumab in combination with idarubicin and cytarabine produced an encouraging response rate (ORR 80%) and OS (median OS 18.5 months) in patients with newly diagnosed AML [168]. The combination therapy of nivolumab and azacitidine was feasible in patients with R/R AML, and the addition of ipilimumab further upregulated the clinical efficacy (ORR 33% vs 44%; median OS 6.4 vs 10.5 months) [169, 170]. And nivolumab maintenance was safe and feasible in high-risk AML patients in CR (1-year CR duration 71%; 1-year OS 86%) [171]. The outcomes of pembrolizumab administered in combined with decitabine or following high-dose cytarabine in R/R AML (ORR 10% and 46%; median OS 7 and 8.9 months, respectively) suggested that immune checkpoint inhibitors after intensive cytotoxic chemotherapy may be a better option [172, 173]. A phase I/Ib study demonstrated the safety and efficacy of ipilimumab monotherapy in AML patients with post-HCT relapse (ORR 32%; 1-year OS 49%) [174]. As for anti-TIM-3 antibody MBG453, the combination therapy with decitabine was safe and well-tolerated and exhibited encouraging preliminary response rates for AML in a phase Ib study (ORR 29% for both newly diagnosed and R/R AML) [175]. However, caution should be paid to checkpoint inhibitors, since exposure can lead to a significantly increased risk of GvHD [168, 174, 176, 177]. Furthermore, the prognostic effect of TIGIT in the bone marrow post-HCT as well as the involvement of CD137-CD137L and CD200-CD200R interactions in immune evasion raise the possibility of attacking other inhibitory receptors with antibodies as potent immunotherapeutic strategies in the near future [53, 178–180].

#### BiKE and TriKE

Bi-specific killer cell engager (BiKE) and tri-specific killer cell engager (TriKE) are the recombinant agents of bivalent and trivalent single-chain variable fragments (scFv), serving as immunologic synapses between NK cells and tumor cells. They retain the specificity of original antibodies and, at the same time, minimize the size of antibodies to increase distribution. CD16-directed BiKE and TriKE trigger NK cell activation through CD16 signaling and against tumor cells with target antigens in a highly efficient manner (Fig. 2c) [181].

Wiernik et al. designed a novel full humanized BiKE that specifically binds to both CD16 and CD33 (CD16 × 33 BiKE). NK cell cytotoxicity and cytokine release were specifically triggered by CD16 × 33 BiKE when cultured with CD33 + AML cell lines and primary AML cells, and the effector functions of NK cells were further enhanced when combined with adisintegrin and metalloprotease-17 (ADAM17) inhibitor which prevents CD16 shedding [182]. Lately, the same research group designed a TriKE by incorporating a novel modified human IL-15 crosslinker into CD16 × 33 BiKE, which provided a signal for NK cell self-sustaining proliferation and activation [183]. A phase I/II clinical trial of CD16 × 33 × IL-15 TriKE (GTB-3550) for the treatment of CD33 + R/R AML is underway (NCT03214666). TriKEs of linking anti-CD16 scFv to either two scFv against the same antigen (such as CD16 × 33 × 33 TriKE) or two scFv against two different antigens (such as CD16 × 33 × 123 TriKE) displayed greater binding affinity and superior NK cell cytotoxic potency toward AML cells compared to BiKE [184, 185]. Since CD33 is abundantly expressed on healthy myeloid cells as well, NKG2DLs, which are leukemia cell-restricted expressed, become promising targets. CD16 × NKG2D BiKE displayed increased affinity to CD16 and induced superior leukemia cell killing compared to the engineered NKG2D-Fc fusion protein [186]. Besides, CD16 × CLL-1 × IL-15 TriKE displayed robust NK cell activity against AML in vitro and in vivo [187]. These molecules constitute attractive candidates for personalized immunotherapy for AML based on preclinical findings.

### Cytokines

Cytokines, including IL-2, IL-12, IL-15, IL-18 and IL-21, play an important role in NK cell proliferation, activation and effector function (Fig. 2e). Ex vivo stimulation with 10 ng/mL IL-2 or 50 ng/mL IL-15 was reported to be optimal for NK cell expansion and enable NK cells of AML patients with recovered function through upregulating activating receptors such as NKp30, NKp46, NKG2C and NKG2D [188–190]. IL-2 monotherapy may not be clinically efficacious in AML patients [191–194]. But, IL-2 in conjunction with histamine dihydrochloride has been proposed as a maintenance therapy in AML, resulting in improved leukemia-free survival [195, 196]. The mechanism of this therapy may partially be the induction of a striking expansion of immunocompetent CD56^bright^ NK cell subpopulations [197]. A phase I study identified IL-15 superagonist complex ALT-803 as a safe agent in the treatment of elderly AML patients who relapsed after HCT and the potential efficacy is expected to be reported (NCT01885897) [198]. And the feasibility of using ALT-803 as an relapse prophylaxis for AML patients after HCT is under assessment (NCT02989844). Furthermore, genetically engineered AML cells with DNA encoding IL-12 or IL-15 have been constructed to reduce toxicities associated with systemic administration of cytokines [199, 200]. A clinical trial (NCT02483312) is ongoing to test engineered AML cells expressing IL-12 in AML patients that cannot have HCT.

Cytokines have also been widely incorporated in the NK cell transfer as a process of ‘priming or arming’ in order to increase NK cell proliferation and expansion. However, the effect is short-lasting and the short-term NK cell persistence within patients might limit their clinical use. Remarkably, NK cells preactivated with a cocktail of cytokines (IL-12, IL-15 and IL-18) exhibited augmented anti-leukemia responses to restimulation for weeks to months regardless of inhibitory KIR-KIR ligand interactions [201–203]. Those cytokine-induced memory-like (CIML) NK cells with adaptive immune properties represent a promising approach to enhancing adoptive NK cell transfer. The first-in-human trial of adoptive transfer of CIML NK cells in elderly patients with R/R AML showed successful induction of remission (ORR 67%) without the cause of CRS, GvHD or neurotoxicity [204, 205]. Patient outcomes were negatively associated with the frequency of CD8α + donor NK cells and the expression of NKG2A on CIML NK cells within patients [205]. Encouraging preliminary data give us confidence on more ongoing early phase clinical trials of CIML NK cells for R/R AML (NCT04354025, NCT02782546, NCT01898793, NCT03068819) [206, 207].

### Drugs with immunomodulatory function

Many anti-tumor drugs have been illustrated with immunomodulatory properties to enhance endogenous NK cell function against AML in recent years (Fig. 2f). Since AML cells resist to NK cell-mediated killing by changing the expression of their surface ligands for various NK cell receptors and these phenotypic defects correlate with clinical outcomes, drugs that are capable of restoring ligand expressions on AML cells can render them more susceptible to NK cell killing [64].

Firstly, hypomethylating agents azacitidine and decitabine can upregulate the expression of NKG2DL on AML cells by reversing epigenetically silenced genes, resulting in enhanced NK cell-mediated immunity through the immune recognition mediated by NKG2D-NKG2DL engagement [208]. They concurrently increase the expression of tissue inhibitor of metalloproteinases-3 (TIMP3), an ADAM17 inhibitor, thus reducing the shedding of soluble NKG2DLs from AML cells [209]. Histone deacetylase inhibitors (trichostatin A and valproic acid), differentiation-promoting drugs (vitamin D3, bryostatin 1 and all-trans-retinoic acid) and hydroxyurea all somehow show the potential of upregulating the expression of NKG2DLs on AML cells, while dinaciclib-treated AML is associated with the downregulation of inhibitory NK ligand HLA-E on AML cells, consequently inducing potent NK cell anti-tumor immunity [208, 210–213]. Then, immunomodulatory drugs lenalidomide and pomalidomide exert anti-leukemia effects both directly and via NK cell-mediated immunostimulatory activities along with downregulation of HLA-class I on AML blasts [214]. The combination therapies containing the aforementioned drugs for AML are widely used in clinical practice and also in clinical trials. Besides, natural compounds or their derivatives such as safrole, α-phellandrene, casticin and ouabain can also promote NK cell activity against AML cells [215–218]. In addition, novel agents with immunomodulatory function were proposed in fundamental researches, providing therapeutic implications in AML. For instance, vascular endothelial growth factor receptor (VEGFR)-3 antagonist restored NK cell cytotoxicity with an increased IFN-γ level [219, 220], and the therapeutic efficacy of adoptive NK cell transfer could be enhanced by a TGF-β receptor kinase inhibitor galunisertib [221]. With the clarification of mechanisms of anti-tumor drugs, combining pharmacological approaches with other NK cell-based immunotherapies may strengthen the efficacy and provide a clinical benefit for AML patients.

## Conclusions and perspectives

Results from current preclinical studies and clinical trials highlight the significant contribution of numerous NK cell-based immunotherapies in activating the reconstitution of NK cells against AML. Adoptive NK cell transfer has expanded the option of cellular immunotherapy as a feasible strategy to induce and maintain remission. Strategies of manipulating adoptively transferred NK cells, such as CAR modification and cytokine induction, may further enhance the therapeutic efficacy. Other strategies, such as immune checkpoint inhibitors, BiKE/TriKE and immunomodulatory drugs, can reverse endogenous NK cell anergy, contributing to an increasing dimensions of utilizing NK cells to fight AML.

There are several advantages in NK cell-based immunotherapy. Firstly, NK cells detect tumor cells through native receptors in a non-MHC-restricted manner and also mediate ADCC, expanding their clinical applications. Secondly, as compared with T-cell therapy, NK-cell therapy has better safety profiles with rare instances of GvHD and CRS due to limited lifespan and distinct cytokines produced [71]. Thirdly, NK cells have the advantage of “off-the-shelf” manufacturing, making it easy to be prepared under good manufacturing practice standards and convenient to universally treat patients with increased speed of administration [222–225]. However, the field of NK cell-based immunotherapy still faces several challenges. In fact, short lifespan of NK cells narrows the therapeutic window, leading to a relatively short duration of response in most patients [88, 90, 95, 226]. Besides, tumors can escape from NK cell cytotoxicity via immunosuppressive tumor microenvironment or by shedding soluble ligands that activate NK receptors [54, 60]. Finally, transduction efficiency of CAR-NK cells is another aspect needed to be improved [227].

In the future, the efficacy of NK cell-based immunotherapy is waiting to be confirmed in large sample sizes and in great detail. The optimal dosage and schedule of adoptive NK cell transfer as well as the feasible sources and manipulation methods for NK cells have yet to be evaluated [228]. It seems logical to combine various NK cell-based immunotherapies to utilize the full potential of NK cells, such as stimulating both target-specific lysis and ADCC effects as well as simultaneously boosting endogenous NK cells and receiving exogenous NK cells [131, 135, 136, 229, 230]. Also, it is reasonable to integrate them with well-established AML treatments or novel agents which may provide synergistic effects and improve clinical response [94]. As for preclinical researches, a better knowledge of the mechanisms of NK cell dysfunction and NK cell-based immunotherapy in AML could broaden the application of NK cells and help the discovery of additional new therapeutic opportunities, including new targets and potential combination therapies. Strategies of wisely using cytokines, such as CMIL NK cells and the transduction of genes encoding cytokines into NK cells, seem to prolong the duration of NK cell persistence in some degree, but more efforts are warranted to figure out approaches to enhance tumor-immune surveillance long term [17, 183, 206, 231]. Taking advantage of multi-omics and information technology, investigation of both donor NK cell-intrinsic and host factors which may contribute to treatment response or resistance can provide an array of biomarkers in donor and patient selection. Overall, there is a bright future in NK cell-based immunotherapy for AML.

## Data Availability

The material supporting the information of this review has been included within the article.

## Abbreviations

ADAM17: A disintegrin and metalloprotease-17
ADC: Antibody-drug conjugate
ADCC: Antibody-dependent cell-mediated cytotoxicity
AE: Adverse event
ALL: Acute lymphoblastic leukemia
allo-HCT: Allogeneic hematopoietic cell transplantation
AML: Acute myeloid leukemia
BiKE: Bi-specific killer cell engager
CAR: Chimeric antigen receptor
CBT: Cord blood transplantation
CD: Cluster of differentiation
cIgTCR: Immunoglobulin-based chimeric T cell receptor
CIML: Cytokine-induced memory-like
CML: Chronic myeloid leukemia
CMV: Cytomegalovirus
CR: Complete remission
CRS: Cytokine release syndrome
CTLA-4: Cytotoxic T lymphocyte-associated antigen-4
CLEC12A: C-type lectin domain family 12, member A
DC: Dendritic cell
DLI: Donor lymphocyte infusion
CLL-1: C-type lectin-like molecule 1
DNAM-1: DNAX accessory molecule-1
FDA: Food and Drug Administration
FLT3: FMS-like tyrosine kinase 3
G-CSF: Granulocyte colony-stimulating factor
GITR: Glucocorticoid-induced TNFR-related protein
GO: Gemtuzumab ozogamicin
GvHD: Graft-versus-host disease
GvL: Graft-versus-leukemia
HCT: Hematopoietic cell transplantation
HLA: Human leukocyte antigen
HSC: Hematopoietic stem cell
HSPC: Hematopoietic stem and progenitor cell
IDO: Indoleamine 2,3 dioxygenase
IFN: Interferon
IL: Interleukin
IL1RAP: IL-1 receptor accessory protein
IL2DT: IL-2 diphtheria toxin
iPSC: Induced pluripotent stem cell
JMML: Juvenile myelomonocytic leukemia
KIR: Killer immunoglobulin-like receptor
LILRB4: Leukocyte immunoglobulin-like receptor subfamily B4
LIR: Leukocyte immunoglobulin-like receptor
LSC: Leukemia stem cell
MACS: Magnetic cell sorting
MDS: Myelodysplastic syndrome
MDSC: Myeloid-derived suppressor cell
MHC: Major histocompatibility complex
MIC: MHC class I chain-related protein
MLL: Mixed lineage leukemia
MPN: Myeloproliferative neoplasm
MRD: Minimal residual disease
MTD: Maximum tolerated dose
NA: Not applicable
NCR: Natural cytotoxicity receptor
NK: Natural killer cell
NKG2A: Natural killer group 2A
NKG2C: Natural killer group 2C
NKG2D: Natural killer group 2D
NKG2DL: NKG2D ligand
NPM1: Nucleophosmin 1
ORR: Overall response rate
PBMC: Peripheral blood mononuclear cell
PD-1: Programmed cell death-1
PD-L1: Programmed cell death ligand-1
PD-L2: Programmed cell death ligand-2
RANKL: Receptor activator for NF-κB ligand
R/R: Relapsed/refractory
scFv: Single chain variable fragment
Siglec: Sialic acid-binding immunoglobulin-like lectin
TAM: Tumor-associated macrophage
TGF: Transforming growth factor
TIGIT: T-cell immunoglobulin and immunoreceptor tyrosine-based inhibitory motif domain
TIM-3: T-cell immunoglobulin domain and mucin domain-3
TIMP3: Tissue inhibitor of metalloproteinases-3
TNF: Tumor necrosis factor
TNFR: Tumor necrosis factor receptor
Treg: Regulatory T cell
TriKE: Tri-specific killer cell engager
UCB: Umbilical cord blood
ULBP: UL16-binding protein
VEGFR: Vascular endothelial growth factor receptor
